# My Experience with the Phylacogens

**Published:** 1916-12

**Authors:** Ferd. D. Bradford

**Affiliations:** Birmingham, Ala.


### MY EXPERIENCE WITH THE PHYLACOGENS.

By Ferd. D. Bradford M. D., Birmingham, Ala.

I shall begin my address by making a very unusual statement, one which you may be inclined to discredit; however, it is made with a clear conscience, and only after careful and thorough study of my cases, for I have had the pleasure of holding all of them under my control and observation throughout the entire treatment. The statement I referred to is this: Out of every 100 cases I have treated with Phylacogens, 100 per cent. have been successful. I know this sounds almost incredible, but I have the case histories to bear me out. I dare say it is a more remarkable success than can be presented by any other practitioner devoting a large part of his practice to urological diseases.

My first acquaintance with this comparatively new weapon in the physician's armamentarium was during my junior year in college. At this time I became greatly interested in what I consider almost a specific, Rheumatism Phylacogen in the treatment of rheumatism. Upon entering practice and hearing both the adverse and commendatory criticisms concerning Phylacogens, I decided to withhold my opinions until I could study the preparations clinically.

I will not burden you with a detailed report of the 100 or more cases, but shall only give one or more from the separate disease complications treated.

I wish to state that I have not used Phylacogens in the treatment of acute specific uncomplicated urethritis, for I have found that none of the gonococcic serums or vaccines have any favorable influence on this malady; it is in the complications and sequelae that Phylacogen plays its important role.

In most of my cases the product was administered subcutaneously but in some the intravenous route was chosen. The subcutaneous method causes less systemic reaction to follow the injection, and permits of larger dosage and thereby quicker results. Idiosyncrasy seems to play almost an unnoticeable part; the slight local reaction being the only inconvenience the patent experiences.

**Case 1.**—O. B., male, age 25, Porto Rican. Subacute specific urethritis and left epididymitis. Five days standing. Urethral discharge very slight as is usual in such cases. Placed a suspensory on at first visit. Administered 2½ cc Gonorrhea Phylacogen the next day, June 10, 1914. Within 24 hours there was a marked decrease in swelling and pain, and the patient reported a good night's rest, the first in several days. Temperature reduced from 102 degrees to 99 degrees. A local dressing was applied to testicle on first visit. On the 12th the second dose of 2½ cc was given. The following morning practically all swelling and pain had disappeared, temperature normal, patient sitting around room, and I advised that he call at my office on the 14th, at which time 2½ cc was given and the dressing changed. All swelling and pain were gone, a slight hardness of the head of the epididymis remaining. On the 16th the last 2½ cc was given against the patient's wishes, he stating he was well. On the 20th all signs of epididymitis having disappeared and the urethral discharge having reappeared, treatment was begun on it, and by July 20th he was discharged, cured. To the present time he has had no further trouble.

**Case 2.**—H. M., aged 29, white, railroad conductor. Acute specific urethritis and right epididymitis. Acute specific urethritis, with slight pain in right testicle. On second day fully developed epididymitis presented itself. All treatment to urethra stopped, patient put at rest except daily visits to my office. Gave 3 cc Gonorrhea Phylacogen. The following day temperature reduced from 101 degrees to 99 degrees, pain and swelling reduced. The patient had suffered a slight systemic and local reaction, but was able to walk to office fairly comfortable. On the following day 3 cc more were given, with a slightly increased systemic reaction and about same local reaction; a marked reduction in pain and swelling, temperature 99 degrees, appetite medium, patient more active. Two days later 4 cc were given and the day following there was complete subsidence of pain and general swelling, temperature was normal, appetite good, the patient assumed nearly normal activity. The usual little knuckle in epididymitis was present with tenderness to touch and the discharge reappeared. Treatment of the urethra was begun and patient advised to allow administration of more Phylacogen. Four injections of 2½ cc were made and the knuckle almost entirely disappeared under this treatment, but the patient, being desirous of marrying in a few months, was anxious to know if I thought further treatment would clear it up. I advised continued treatment and four more injections of 2½ cc were administered, one every other day, and this succeeded in completely obliviating the condition. During this time the urethritis had also responded and at present time, after severe tests, patient is in best of health and plans marriage soon. He thought he would be compelled to enter a hospital, but I assured him that under my new and improved method, this would be unnecessary. He resumed work in two weeks after beginning treatment.

**Case 3**—T. S., aged 22, male, colored, waiter. Gonorrheal arthritis. When first seen patient was seeking aid for chancre of meatus and penis. The secondard manifestations were present and diagnosis of lues affirmed. All lesions were healed and patient discharged under specific constitutional treatment. A good balsamic was also given along with other treatment to antisepticize the urethral tract. About two weeks after discharging patient, he returned complaining of swelling and pain in both feet which kept him from work. I increased the potassium iodide in his mixed treatment and applied local treatment, but the condition only improved temporarily and the next joints to show trouble were both ankles, then followed the knee, hip, shoulder and wrist joints. Through all this the patient was undergoing great suffering and had to be handled as a baby. After a few days heroic effort with routine treatment, I decided to administer Gonorrhea Phylacogen in my usual way, and on May 27th the first injection of 3(cc) was given. Systemic and local reaction followed in about six hours. The next day the patient reported a good night and more comfort than at any time in the last week. On the 29th the second injection of 3 (cc) was given with less systemic reaction, and the following day he was able to turn himself over in bed and eothrwise attend himself. On the 31st 4 (cc) were given, and on June 3rd the patient visited my office, walking to and from the street car. He resumed work on the 7th. On the 10th he returned with a marked artritic condition of right wrist and I immediately resumed the Phylacogen treatment in my usual way, giving 100 (cc) in all. In three days all symtoms had practically cleared and he was at work again, and now is completely relieved. I still have him under specific treatment· This case presented some puzzling features and worried me much, but I determined that there was a mixed infection present and that the meatal chancre obscured the specific urethritis only to have it manifest itself in an arthritis. I therefore selected the Gonorrhea Phylacogen instead of the Rheumatism Phylacogen.

Herein lies the success of Phylacogen therapy—careful diagnosis.

**Case 4.**—C. D., aged 28, expressman. Specific urethritis and gonorrheal arthritis. Feb. 1st, specific urethritis. Treated same until all discharge had cleared up and patient had resumed coitus. A week later there was an apparent acute return and in about eight days he complained of pain and swelling in both ankles and was compelled to stop work. Gonorrhea Phylacogen was ordered and the first injection of 2 (cc) made. In the meantime he had taken to his bed, other articulations became involved, temperature 102 degrees, and general discomfiture. After the first injection general improvement was noted· Three more injections were given and in six days he was able to resume work, but in a few days his shoulders were troubling him so much that I administered another 10 cc. Today there are no signs of the arthritis and have not been since April 1st. The specific urethritis was not influenced by the Phylacogen, but the arthritis cleared up beautifully.

The above case will give you a fair idea as to my results with Gonorrhea Phylacogen. However, before closing I want to state a few words as to the composition of this product.

Gonorrhea Phylacogen is a sterile aqueous solution of bacterial derivatives from cultures of the Mocrococcus Gonorrhea, and in less proportion of other pathogenic bacteria, viz.: Strep. rheumaticus, Strep. pyogenes, Strep. erysipelatis, Staph. pyogenes, B. pyocyaneus, B. coli communis, B. diptheriae and Diplococcus pneumoniae, numerous strains from a variety of sources being employed. This complex constitution is based upon the opinion which Dr. Schafer, the originator of Phylacogen therapy, holds in common with many practitioners of wide repute, that most chronic infections are complicated in character, and though one organism may predominate, the pathologic process which it engenders is intensified by other organisms present in the system, which assume pathologic significance when physiologic resistance is below par.

Contra-indications: Intravenous injections should never be given in terminal cases, nor to cases with cardiac development, pronounced arteriosclerosis or nephritis· There is no contra-indication to the subcutaneous method unless it be nephritis, though in long standing heart lesions of a grave nature, great care should be exercised. The treatment should be carried out cautiously in alcoholics. In my practice injections are given every other day as a rule.

I have kept accurate records of all cases I have treated and have carefully observed the results of Phylacogen treatment. As a result, I must confess my utmost faith in this therapy and trust my experience will prove helpful to other members of the profession.

